# Rapid metagenomic identification of viral pathogens in clinical samples by real-time nanopore sequencing analysis

**DOI:** 10.1186/s13073-015-0220-9

**Published:** 2015-09-29

**Authors:** Alexander L. Greninger, Samia N. Naccache, Scot Federman, Guixia Yu, Placide Mbala, Vanessa Bres, Doug Stryke, Jerome Bouquet, Sneha Somasekar, Jeffrey M. Linnen, Roger Dodd, Prime Mulembakani, Bradley S. Schneider, Jean-Jacques Muyembe-Tamfum, Susan L. Stramer, Charles Y. Chiu

**Affiliations:** Department of Laboratory Medicine, University of California, San Francisco, CA 94107 USA; UCSF-Abbott Viral Diagnostics and Discovery Center, San Francisco, CA 91407 USA; Institut National de Recherche Biomédicale, Kinshasa, Democratic Republic of the Congo Africa; Hologic, Inc, Bedford, MA 01730 USA; American Red Cross, Gaithersburg, MD 2087 USA; Metabiota, Inc, San Francisco, CA 94104 USA; Department of Medicine, Division of Infectious Diseases, University of California, San Francisco, CA USA

## Abstract

We report unbiased metagenomic detection of chikungunya virus (CHIKV), Ebola virus (EBOV), and hepatitis C virus (HCV) from four human blood samples by MinION nanopore sequencing coupled to a newly developed, web-based pipeline for real-time bioinformatics analysis on a computational server or laptop (MetaPORE). At titers ranging from 10^7^–10^8^ copies per milliliter, reads to EBOV from two patients with acute hemorrhagic fever and CHIKV from an asymptomatic blood donor were detected within 4 to 10 min of data acquisition, while lower titer HCV virus (1 × 10^5^ copies per milliliter) was detected within 40 min. Analysis of mapped nanopore reads alone, despite an average individual error rate of 24 % (range 8–49 %), permitted identification of the correct viral strain in all four isolates, and 90 % of the genome of CHIKV was recovered with 97–99 % accuracy. Using nanopore sequencing, metagenomic detection of viral pathogens directly from clinical samples was performed within an unprecedented <6 hr sample-to-answer turnaround time, and in a timeframe amenable to actionable clinical and public health diagnostics.

**Electronic supplementary material:**

The online version of this article (doi:10.1186/s13073-015-0220-9) contains supplementary material, which is available to authorized users.

## Background

**Fig. 1:**
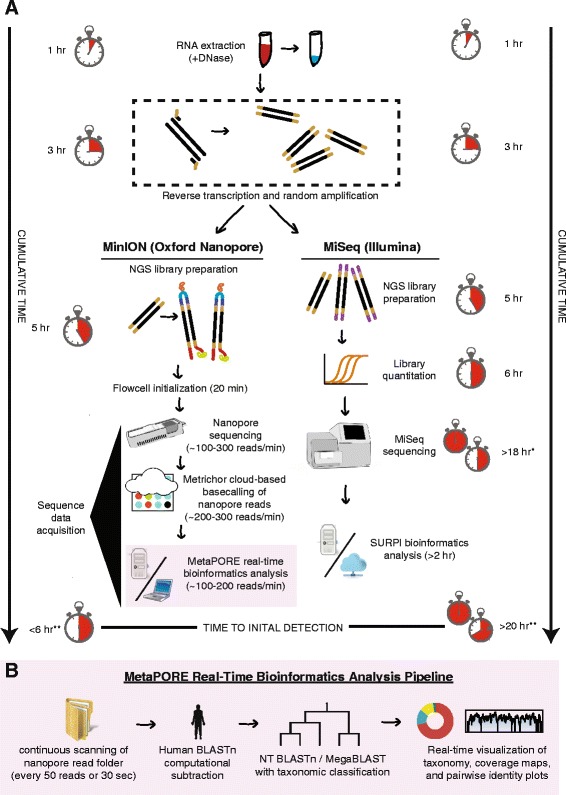
Metagenomic sequencing workflow for MinION nanopore sequencing compared to Illumina MiSeq sequencing. **a** Overall workflow. **b** Steps in the MetaPORE real-time analysis pipeline. The turnaround time for sample-to-detection nanopore sequencing, defined here as the cumulative time taken for nucleic acid extraction, reverse transcription, library preparation, sequencing, MetaPORE bioinformatics analysis, and pathogen detection, was under 6 hr, while Illumina sequencing took over 20 hr. The time differential is accounted for by increased times for library quantitation, sequencing, and bioinformatics analysis with the Illumina protocol. *Assumes a 12-hr 50-bp single-end MiSeq run of ~12–15 million reads, with 50 bp the minimum estimated read length needed for accurate pathogen identification. **Denotes estimated average SURPI bioinformatics analysis run length for MiSeq data [19]. The stopwatch is depicted as a 12-hr clock

Acute febrile illness has a broad differential diagnosis and can be caused by a variety of pathogens. Metagenomic next-generation sequencing (NGS) is particularly attractive for diagnosis and public health surveillance of febrile illness because the approach can broadly detect viruses, bacteria, and parasites in clinical samples by uniquely identifying sequence data [1, 2]. Although currently limited by sample-to-answer turnaround times typically exceeding 20 hr (Fig. 1a), we and others have reported that unbiased pathogen detection using metagenomic NGS can generate actionable results in timeframes relevant to clinical diagnostics [3–6] and public health [7, 8]. However, timely analysis using second-generation platforms such as Illumina and Ion Torrent has been hampered by the need to wait until a sufficient read length has been achieved for diagnostic pathogen identification, as sequence reads for these platforms are generated in parallel and not in series.

Nanopore sequencing is a third-generation sequencing technology that has two key advantages over second-generation technologies – longer reads and the ability to perform real-time sequence analysis. To date, the longer nanopore reads have enabled scaffolding of prokaryotic and eukaryotic genomes and sequencing of bacterial and viral cultured isolates [9–13], but the platform’s capacity for real-time metagenomic analysis of primary clinical samples has not yet been leveraged. As of mid-2015, the MinION nanopore sequencer is capable of producing at least 100,000 sequences with an average read length of 5 kb, in total producing up to 1 Gb of sequence in 24 hr on one flow cell [14]. Here we present nanopore sequencing for metagenomic detection of viral pathogens from clinical samples with a sample-to-answer turnaround time of under 6 hr (Fig. 1a). We also present MetaPORE, a real-time web-based sequence analysis and visualization tool for pathogen identification from nanopore data (Fig. 1b).

## Methods

### Ethics statement

The chikungunya virus (CHIKV) plasma sample was collected from a donor from Puerto Rico, who provided written consent for use of samples and de-identified clinical metadata in medical research [15]. For the Ebola virus (EBOV) samples, patients provided oral consent for collection and analysis of their blood, as was the case for previous outbreaks [16, 17]. Consent was obtained either at the homes of patients or in hospital isolation wards by a team that included staff members of the Ministry of Health in the Democratic Republic of the Congo (DRC). The hepatitis C virus (HCV) sample was a banked aliquot from a patient with known hepatitis C infection at the University of California, San Francisco (UCSF), and sequence analysis was performed under a waiver of consent granted by the UCSF Institutional Review Board.

### MAP program

Since July 2014, our lab has participated in the MinION Access Program (MAP), an early access program for beta users of the Oxford Nanopore MinION. Program participants receive free flow cells and library preparation kits for testing and validation of new protocols and applications on the MinION platform. During our time in the MAP program, we have seen significant progress in sequencing yield, although the quality of flow cells has varied considerably and individual read error rates remain high (Table 1).

**Table 1:** Flow cell run data

Exp/sample	Flow cell #	Run #	# of active pores	Run time (min)	Total reads	Pass reads	Fail reads	Pass/fail rate	Target virus	# of aligned reads	Avg read length [range] (bp)	Avg read error rate^a^
Chik1	1	First run	345	138	19,452	5,139	14,313	35.9 %	CHIKV	556	455 [126–1477]	20.6 % (8–49 %)
Ebola1	1	First run	105	1022	13,090	1,831	11,259	16.3 %	EBOV	41	358 [220–672]	22.0 % (12–43 %)
HepC1	2	First run	171	122	10,305	729	9,877	7.4 %	HCV	6	572 [318–792]	33.1 % (24–46 %)
HepC1	2	Reload #1	293	192	26,626	2,155	25,758	8.4 %	HCV			
HepC1	2	Reload #2	256	298	32,212	1,207	31,289	3.9 %	HCV			
HepC1	2	Reload #3	214	156	14,805	287	14,275	2.0 %	HCV			
Ebola2	3	First run	397	79	28,651	1,537	27,114	5.7 %	EBOV	593	456 [189–1430]	22.3 % (8–48 %)
Ebola2	3	Reload #1	426	222	95,861	2,899	92,962	3.1 %	EBOV			
Ebola2	3	Reload #2	380	1091	166,524	1,539	164,985	0.9 %	EBOV			
Ebola2	3	Reload #3	200	1357	44,272	34	44,238	0.1 %	EBOV			
TOTAL					451,798	17,357	436,070	4.0 %			452 [126–1477]	24.3 % (8–49 %)

*Exp*experiment, *CHIKV* chikungunya virus, *EBOV* Ebola virus, *HCV*, hepatitis C virus

^a^Based on average pairwise identity of aligned viral reads to the most closely matched reference sequence

### Nucleic acid extraction

Frozen surplus plasma samples were collected during the peak weeks of the 2014 CHIKV outbreak in Puerto Rico from blood donors [15], and were de-identified prior to inclusion in the study. Total nucleic acid was extracted from 400 μL of a CHIKV-positive plasma sample (Chik1) inactivated in a 1:3 ratio of TRIzol LS (Life Technologies, Carlsbad, CA, USA) at the American Red Cross prior to shipping to UCSF. The Direct-zol RNA MiniPrep Kit (Zymo Research, Irvine, CA, USA) was used for nucleic acid extraction, including on-column treatment with Turbo DNAse (Life Technologies) for 30 min at 37 °C to deplete human host genomic DNA.

For the EBOV samples, total nucleic acid was extracted using the QIAamp Viral RNA kit (Qiagen, Valencia, CA, USA) from 140 μL of whole blood from two patients with suspected Ebola hemorrhagic fever during a 2014 outbreak in the DRC (Ebola1 and Ebola2). RNA was extracted at Institut National de Recherche Biomédicale in Kinshasa, DRC, preserved using RNAstable (Biomatrica, San Diego, CA, USA), and shipped at room temperature to UCSF. Upon receipt, the extracted RNA sample was treated with 1 μL Turbo DNase (Life Technologies), followed by cleanup using the Direct-zol RNA MiniPrep Kit (Zymo Research).

For the HCV sample, an HCV-positive serum sample at a titer of 1.6 × 10^7^ copies/mL (HepC1) was diluted to 1 × 10^5^ copies/mL using pooled negative serum. Total nucleic acid was then extracted from 400 μL of serum using the EZ1 Viral RNA kit, followed by treatment with Turbo DNase for 30 min at 37 °C and cleanup using the RNA Clean and Concentrator Kit (Zymo Research).

### Molecular confirmation of viral infection

A previously reported TaqMan quantitative reverse-transcription polymerase chain reaction (qRT-PCR) assay targeting the EBOV NP gene was used for detection of EBOV and determination of viral load [18]. The assay was run on a Stratagene MX300P real-time PCR instrument and performed using the TaqMan Fast Virus 1-Step Master Mix (Life Technologies) in 20 μL total reaction volume (5 μL 4× TaqMan mix, 1 μL sample extract), with 0.75 μM of each primer (F565 5′-TCTGACATGGATTACCACAAGATC-3′, R640 5′-GGATGACTCTTTGCCGAACAATC-3′) and 0.6 μM of the probe (p597S 6FAM-AGGTCTGTCCGTTCAA-MGBNFQ). Conditions for the qRT-PCR were modified as follows: 50 °C for 10 min and 95 °C for 20 s followed by 45 cycles of 95 °C for 3 s plus 60 °C for 30 s. Viral copy number was calculated by standard curve analysis using a plasmid vector containing the EBOV amplicon. The first EBOV sample analyzed by nanopore sequencing (Ebola1) corresponded to the Ebola virus/*H.sapiens*-wt/COD/2014/Lomela-Lokolia16 strain, while the second Ebola sample (Ebola2) corresponded to the Ebola virus/*H.sapiens*-wt/COD/2014/Lomela-LokoliaB11 strain. The CHIKV-positive sample was identified and quantified using a transcription-mediated amplification assay (Hologic, Bedford, MA, USA) as previously described [15]. HCV was quantified using the Abbott RealTime RT-PCR assay, approved by the Food and Drug Administration, as performed in the UCSF Clinical Microbiology Laboratory on the Abbott Molecular m2000 system.

### Construction of metagenomic amplified cDNA libraries

To obtain ≥1 μg of metagenomic complementary DNA (cDNA) for the library required for the nanopore sequencing protocol, randomly amplified cDNA was generated using a primer-extension pre-amplification method (Round A/B) as described previously [19–21]. Of note, this protocol has been extensively tested on clinical samples for metagenomic pan-pathogen detection of DNA and RNA viruses, bacteria, fungi, and parasites [4, 6, 19, 21, 22]. Briefly, in Round A, RNA was reverse-transcribed with SuperScript III Reverse Transcriptase (Life Technologies,) using Sol-PrimerA (5′-GTTTCCCACTGGAGGATA-N_9_-3′), followed by second-strand DNA synthesis with Sequenase DNA polymerase (Affymetrix, Santa Clara, CA, USA). Reaction conditions for Round A were as follows: 1 μL of Sol-PrimerA (40 pmol/μL) was added to 4 μL of sample RNA, heated at 65 °C for 5 min, then cooled at room temperature for 5 min. Then 5 μL of SuperScript Master Mix (2 μl 5× First-Strand Buffer, 1 μL water, 1 μL 12.5 mM dNTP mix, 0.5 μL 0.1 M DTT, 0.5 μL SS III RT) was added and incubated at 42 °C for 60 min. For second strand synthesis, 5 μL of Sequenase Mix #1 (1 μL 5× Sequenase Buffer, 3.85 μL ddH_2_O, 0.15 μL Sequenase enzyme) was added to the reaction mix and incubated at 37 °C for 8 min, followed by addition of Sequenase Mix #2 (0.45 μl Sequenase Dilution Buffer, 0.15 μl Sequenase Enzyme) and there was a second incubation at 37 °C for 8 min. Round B reaction conditions were as follows: 5 μL of Round A-labeled cDNA was added to 45 μL of KlenTaq master mix per sample (5 μL 10× KlenTaq PCR buffer, 1 μL 12.5 mM dNTP, 1 μL 100 pmol/μL Sol-PrimerB (5′-GTTTCCCACTGGAGGATA-3′), 1 μL KlenTaq LA (Sigma-Aldrich, St Louis, MO), 37 μL ddH_2_O). Reaction conditions for the PCR were as follows: 94 °C for 2 min; 25 cycles of 94 °C for 30 s, 50 °C for 45 s, and 72 °C for 60 s, followed by 72 °C for 5 min.

### Preparation of nanopore sequencing libraries

Amplified cDNA from Round B was purified using AMPure XP beads (Beckman Coulter, Brea, CA), and 1 μg DNA was used as input into Oxford Nanopore Genomic DNA MAP-003 Kits (Chik1, Ebola1) or MAP-004 Kits (HepC1, Ebola2) for generation of MinION Oxford Nanopore-compatible libraries [9, 11]. Briefly, the steps include: (1) addition of control lambda phage DNA, (2) end-repair with the NEBNext End Repair Module, (3) 1× AMPure purification, (4) dA-tailing with the NEBNext dA-tailing Module, (5) ligation to protein-linked adapters HP/AMP (Oxford Nanopore Technologies, Oxford, UK) using the NEBNext QuickLigation Module for 10 min at room temperature, (6) purification of ligated libraries using magnetic His-Tag Dynabeads (Life Technologies), and (7) elution in 25 μL buffer (Oxford Nanopore Technologies). Lambda phage DNA was not added during preparation of the Ebola2 sample library.

### Nanopore sequencing

Nanopore libraries were run on an Oxford Nanopore MinION flow cell after loading 150 μL sequencing mix (6 μL library, 3 μL fuel mix, 141 μL buffer) per the manufacturer’s instructions. The Chik1 and Ebola1 samples were run consecutively on the same flow cell, with an interim wash performed using Wash-Kit-001 (Oxford Nanopore).

### Illumina sequencing

For the Chik1 and Ebola1 samples, amplified Round B cDNA were purified using AMPure XP beads (Beckman Coulter) and 2 ng used as input into the Nextera XT Kit (Illumina). After 13 cycles of amplification, Illumina library concentration and average fragment size were determined using the Agilent Bioanalyzer. Sequencing was performed on an Illumina MiSeq using 150 nucleotide (nt) single-end runs and analyzed for viruses using either the MetaPORE or SURPI computational pipeline (UCSF) [19].

### MetaPORE bioinformatics pipeline

We developed a custom bioinformatics pipeline for real-time pathogen identification and visualization from nanopore sequencing data (MetaPORE) (Fig. 1b), available under license from UCSF at [23]. The MetaPORE pipeline consists of a set of Linux shell scripts, Python programs, and JavaScript/HTML code, and was tested and run on an Ubuntu 14.10 computational server with 64 cores and 512 GB memory. In addition, MetaPORE was tested and run on a laptop (Ubuntu 14.10, eight hyper-threaded cores, 32 GB RAM). On the laptop, to maximize sensitivity while still retaining the speed necessary for real-time analysis and web-based visualization, MetaPORE can either (1) restrict the reference database for nucleotide BLAST (BLASTn) alignment to viral sequences or (2) use the faster MegaBLAST instead of the BLASTn algorithm at word sizes ranging from 11 to 28 to align nanopore reads to all of the National Center for Biotechnology Information (NCBI) nucleotide collection database (NT database). Running MegaBLAST to NT at a word size of 16 was found to detect ~85 % of nanopore CHIKV reads (*n* = 196) with an ~8× speedup in processing time relative to BLASTn, or 100 % of EBOV reads (*n* = 98) with an ~5× speedup (Additional file 1: Table S1). Overall, speeds of MegaBLAST to NT alignment at a word size of 16 versus BLASTn to the viral database were slower but comparable (Additional file 2: Table S2).

Raw FAST5/HDF files from the MinION instrument are base-called using the Metrichor 2D Basecalling v1.14 pipeline (Metrichor). The MetaPORE pipeline continually scans the Metrichor download directory for batch analysis of downloaded sequence reads. For each batch of files (collected every time 200 reads are downloaded in the download directory, or ≥2 min of elapsed time, whichever comes first), the 2D read or either the template or complement read, depending on which is of higher quality, is converted into a FASTQ file using HDF5 Tools [24]. The *cutadapt* program is then used to trim Sol-PrimerB adapter sequences from the ends of the reads [25]. Next, the BLASTn aligner is used to subtract host reads computationally [19, 26], aligning to the human fraction of the NT database at word size 11 and e-value cutoff of 10^-5^. The remaining, non-human reads are then aligned by BLASTn (on a 64-core server) or MegaBLAST (on a laptop) to the entire NT database, using the same parameters. Alternatively, the remaining reads can be aligned on a laptop using BLASTn to just the viral fraction of the NT database, followed by BLASTn alignment of the viral reads to the NT database to verify that they are correctly identified. For each read, the single best match by e-value is retained, and the NCBI GenBank gene identifier assigned to the best match is then annotated by taxonomic lookup of the corresponding lineage, family, genus, and species [19].

It has been reported that the LAST alignment algorithm [27] may be more sensitive for nanopore read identification [12, 28]. However, LAST was originally developed for genome-scale alignments, and not for huge databases such as the NT database. To date, it has only been used to align nanopore reads to individual reference sequences [12, 28]. We attempted to use the LAST software to align nanopore reads to the NT database (June 2014, ~60 Gb in size). LAST automatically created multiple formatted database volumes (*n* > 20), each approximately 24 Gb, to encompass all of the NT database. As the run time for loading each volume into memory was just under 2 minutes, resulting in a >40 minutes overhead time, LAST was considered to be impractical for real-time metagenomic sequencing analysis on a single server or laptop.

For real-time visualization of results, a graphical user interface was developed for the MetaPORE pipeline. A live taxonomic count table is displayed as a donut chart using the CanvasJS graphics suite [29], with the chart refreshing every 30 s (Additional file 3). For each viral species detected, the top hit is chosen to be the reference sequence (GenBank identifier) in the NT database assigned to that species with the highest number of aligned reads, with priority given to reference sequences in the following order: (1) complete genomes, (2) complete sequence, or (3) partial sequences or individual genes. Coverage maps are generated by mapping all aligned viral species reads to the top hit reference sequence using LASTZ v1.02 [30], with interactive visualization provided using a custom web program that accesses the HighCharts JavaScript library [31]. A corresponding interactive pairwise identity plot is generated using SAMtools [32] to calculate the consensus FASTA sequence from the coverage map, followed by pairwise 100-bp sliding-window comparisons of the consensus to the reference sequence using the BioPython implementation of the Needleman–Wunsch algorithm [33, 34]. For comparison, the MetaPORE pipeline was also run on a subset of 100,000 reads from parallel Illumina MiSeq data corresponding to the Chik1, Ebola1, and Ebola2 samples.

### Phylogenetic analysis

The overall CHIKV phylogeny consisted of all 188 near-complete or complete genome CHIKV sequences available in the NT database as of March 2015. A subphylogeny, including the MiSeq- and nanopore-sequenced Puerto Rico strain PR-S6 presented here and previously [15], as well as additional Caribbean CHIKV strains and other representative members of the Asian-Pacific clade, was also analyzed. The EBOV phylogeny consisted of the newly MiSeq- and nanopore-sequenced Ebola strain Lomela-LokoliaB11 from the 2014 DRC outbreak [17], as well as other representative EBOV strains, including strains from the 2014–2015 West African outbreak [8, 35]. Sequences were aligned using the MAFFT algorithm [36], and phylogenetic trees were constructed using the MrBayes algorithm [37] in the Geneious software package [38].

### Data availability

Nanopore and MiSeq sequencing data corresponding to non-human reads identified by MetaPORE, along with sample metadata, have been submitted to NCBI under the following GenBank Sequence Read Archive (SRA) accession numbers: Ebola virus/H.sapiens-wt/COD/2014/Lomela-Lokolia16 [SRA:SRP057409], Ebola virus/H.sapiens-wt/COD/2014/Lomela-LokoliaB11 [SRA:SRS933322], Chik1 [SRA:SRP057410] and HepC1 [SRA:SRP057418]. Sequence reads were additionally filtered for exclusion of human sequences by both BLASTn alignment at an e-value cutoff of 10^-5^ and Bowtie2 high-sensitivity local alignment to the human hg38 reference database.

## Results

### Example 1: Nanopore sequencing of high-titer chikungunya virus (Flow cell #1)

**Fig. 2:**
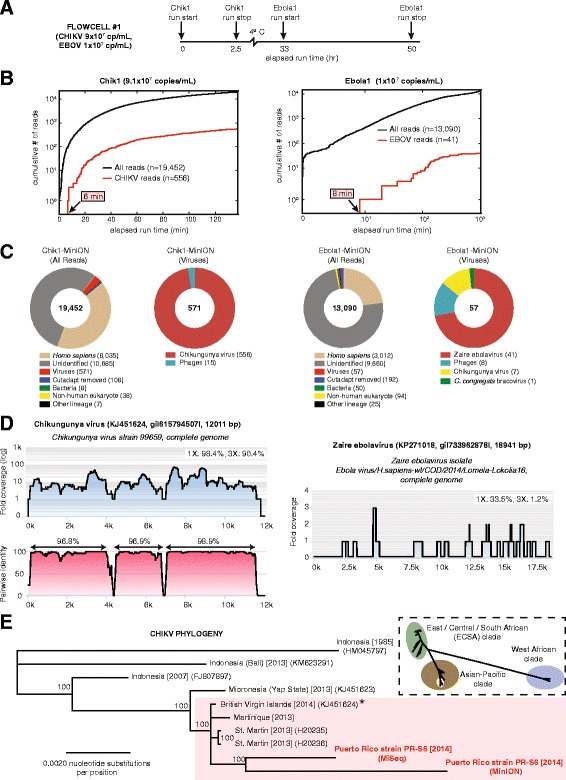
Metagenomic identification of CHIKV and EBOV from clinical blood samples by nanopore sequencing. **a** Time line of sequencing runs on flow cell #1 with sample reloading, plotted as a function of elapsed time in hours since the start of flow cell sequencing. **b** Cumulative numbers of all sequenced reads (*black line*) and target viral reads (*red line*) from the Chik1 run (*left panel*) and Ebola1 run (*right panel*), plotted as a function of individual sequencing run time in minutes. **c** Taxonomic donut charts generated using the MetaPORE bioinformatics analysis pipeline from the Chik1 run (*left panel*) and Ebola1 run (*right panel*). The total number of reads analyzed is shown in the center of the donut. **d** Coverage plots generated in MetaPORE by mapping reads aligning to CHIKV (*left*, Chik1 run) or EBOV (*right*, Ebola1 run) to the closest matching reference genome (**(e)**, *asterisk*). A corresponding pairwise identity plot is also shown for CHIKV, for which there is sufficient coverage. **e** Whole-genome phylogeny of CHIKV. Representative CHIKV genome sequences from the Asian-Pacific clade, including the Puerto Rico PR-S6 strain recovered by nanopore and MiSeq sequencing, or all available 188 near-complete or complete CHIKV genomes (*inset*), are included. Branch lengths are drawn proportionally to the number of nucleotide substitutions per position, and support values are shown for each node. were was analyzed in MetaPORE on a 64-core Ubuntu Linux server using the June 2014 and January 2015 NT databases as the reference databases for the CHIKV and EBOV samples, respectively

To test the ability of nanopore sequencing to identify metagenomic reads from a clinical sample, we first analyzed a plasma sample harboring high-titer CHIKV and previously sequenced on an Illumina MiSeq platform (Fig. 2a) [15]. The plasma sample corresponded to an asymptomatic blood donor who had screened positive for CHIKV infection during the 2014 outbreak in Puerto Rico (strain PR-S6), with a calculated viral titer of 9.1 × 10^7^ copies/mL.

**Fig. 3:**
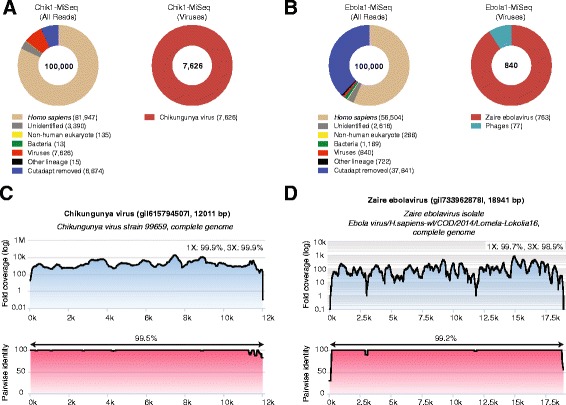
MetaPORE analysis of Illumina MiSeq data from samples containing CHIKV and EBOV. Taxonomic donut charts were generated from Illumina MiSeq data corresponding to the Chik1 run (**a**) and Ebola1 run (**b**) using the MetaPORE bioinformatics analysis pipeline. The total number of MiSeq reads analyzed is shown in the center of the donut. Note that given computational time constraints, only a subset of reads (n = 100,000) was analyzed using MetaPORE. Coverage and pairwise identity plots were generated from MiSeq CHIKV reads from the Chik1 sample (248,677 of 3,235,099 reads, 7.7 %) (**c**), or EBOV reads from the Ebola1 sample (20,820 of 2,743,589 reads, 0.76 %)  (**d**), identified using SURPI analysis and LASTZ mapping {Harris, 2007 #34} at an e-value of 10-5 to the closest matching reference genome. Data were analyzed in MetaPORE on a 64-core Ubuntu Linux server using the June 2014 and January 2015 NT databases as the reference databases for the CHIKV and EBOV samples, respectively.

A read aligning to CHIKV, the 96th read, was sequenced within 6 min (Fig. 2b, left panel) and detected by BLASTn alignment to the NT database within 8 min of data acquisition, demonstrating an overall sample-to-detection turnaround time of <6 hr (Fig. 1). After early termination of the sequencing run at the 2 hr 15 min time point, 556 of 19,452 total reads (2.8 %) were found to align to CHIKV (Fig. 2b, c, left panels). The individual CHIKV nanopore reads had an average length of 455 bp (range 126–1477 bp) and average percentage identity of 79.4 % to the most closely matched reference strain, a CHIKV strain from the neighboring British Virgin Islands (KJ451624), corresponding to an average nanopore read error rate of 20.6 % (range 8–49 %) (Table 1). When only high-quality 2D pass reads were included, 346 of 5139 (6.7 %) reads aligned to CHIKV, comparable to the proportion of CHIKV reads identified by corresponding metagenomic sequencing on the Illumina MiSeq (7.6 % by MetaPORE analysis of 100,000 reads; Fig. 3a, left panel).

Mapping of the 556 nanopore reads aligning to CHIKV to the assigned reference genome (KJ451624) showed recovery of 90 % of the genome at 3× coverage and 98 % at 1× coverage (Fig. 2d, left panel). Notably, despite high individual read error rates, 97–99 % identity to the reference genome (KJ451624) was achieved across contiguous regions with at least 3× coverage. Furthermore, phylogenetic analysis revealed co-clustering of the CHIKV genomes independently assembled from MinION nanopore or Illumina MiSeq reads (Fig. 2d, left panel and Fig. 3b, left panel) on the same branch within the Caribbean subclade (Fig. 2e). Overall, a large proportion of reads (55 %) in the error-prone nanopore data remained unidentifiable, while other aligning reads aside from CHIKV corresponded to human, lambda phage control spike-in, uncultured bacterial, or other eukaryotic sequences (Fig. 2c, left panel).

### Example 2: Nanopore sequencing of high-titer Ebola virus (Flow cell #1)

We next attempted to replicate our metagenomic detection result on the nanopore sequencer with a different virus by testing a whole blood sample from a patient with Ebola hemorrhagic fever during the August 2014 outbreak in the DRC (Ebola1, strain Lomela-Lokolia16) [17]. To conserve flow cells, the same nanopore flow cell used to run the Chik1 sample was washed and stored overnight at 4 °C, followed by nanopore sequencing of the Ebola1 sample (viral titer of 1.0 × 10^7^ copies/mL by real-time qRT-PCR) (Fig. 2b, right panel). Only 41 of 13,090 nanopore reads (0.31 %) aligned to EBOV (Fig. 2c, right panel), comparable to the percentage of reads obtained for Illumina MiSeq (0.84 % by MetaPORE analysis of 100,000 reads; Fig. 3a, right panel). The decrease in relative number and percentage of target viral nanopore reads in the Ebola1 sample relative to the Chik1 sample is consistent with the lower levels of viremia (1.0 × 10^7^ versus 9.1 × 10^7^ copies/mL) and higher host background (whole blood versus plasma). Nonetheless, the first read aligning to EBOV was detected in a similar timeframe as in the Chik1 sample, sequenced within 8 min and detected within 10 min of data acquisition. EBOV nanopore reads were 359 bp in length on average (range 220–672 nt), with an average error rate of 22 % (range 12–43 %) (Table 1). However, despite these error rates, the majority of Ebola nanopore sequences (31 of 41, 76 %) were found to align to the correct strain, Lomela-Lokolia16, as confirmed by MiSeq sequencing (Fig. 2d, right panel and Fig. 3b, right panel).

Despite washing the flow cell between the two successive runs, seven CHIKV reads were recovered during the Ebola1 library sequencing, suggesting the potential for carryover contamination. CHIKV reads were not present in the corresponding Illumina MiSeq Ebola1 run (Fig. 3a, right panel), confirming that the source of the contamination originated from the Chik1 nanopore library, which was run on the same flow cell as and just prior to the Ebola1 library.

### Example 3: Nanopore sequencing of moderate-titer hepatitis C virus (Flow cell #2)

**Fig. 4:**
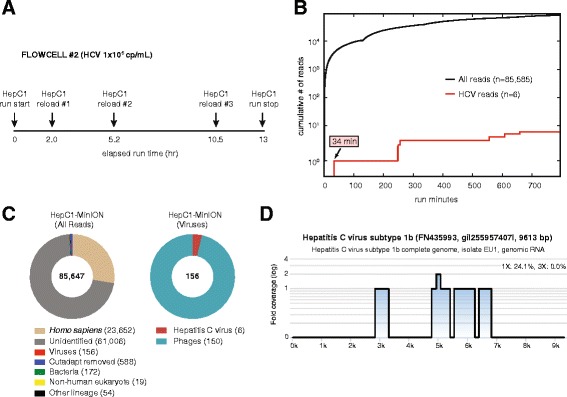
Metagenomic identification of HCV from a clinical serum sample by nanopore sequencing. **a** Time line of sequencing runs on flow cell #2 with HepC1 sample reloading, plotted as a function of elapsed time in hours since the start of flow cell sequencing. **b** Cumulative number of all sequenced reads (*black line*) and HCV viral reads (*red line*), plotted as a function of individual sequencing run time in minutes. **c** Taxonomic donut charts generated using the MetaPORE bioinformatics analysis pipeline. The total number of reads analyzed is shown in the center of the donut. **d** Coverage and pairwise identity plots generated in MetaPORE by mapping reads aligning to HCV to the closest matching reference genome. Data were analyzed in MetaPORE on a 64-core Ubuntu Linux server using the January 2015 NT reference database

Our previous experiments revealed both the total number of metagenomic reads and proportion of target viral reads at a given titer that could be obtained from a single MinION flow cell, and showed that the proportion of viral reads obtained by metagenomic nanopore and MiSeq sequencing was comparable. Thus, we projected that the minimum concentration of virus that could be reproducibly detected using our current metagenomic protocol would be 1 × 10^5^ copies/mL. An HCV-positive clinical sample (HepC1) was diluted in negative control serum matrix to a titer of 1 × 10^5^ copies/mL and processed for nanopore sequencing using an upgraded library preparation kit (MAP-004). After four consecutive runs on the same flow cell with repeat loading of the same metagenomic HepC1 library (Fig. 4a), a total of 85,647 reads were generated, of which only six (0.0070 %) aligned to HCV (Fig. 4b). Although the entire series of flow cell runs lasted for >12 hr, the first HCV read was sequenced within 34 min, enabling detection within 36 min of data acquisition. Given the low titer of HCV in the HepC1 sample and hence low corresponding fraction of HCV reads in the nanopore data, the vast majority (96 %) of viral sequences identified corresponded to the background lambda phage spike-in (Fig. 4c). Importantly, although nanopore sequencing identified only six HCV reads, all six reads aligned to the correct genotype, genotype 1b (Fig. 4d).

### Example 4: Nanopore sequencing of high-titer Ebola virus with real-time MetaPORE analysis (Flow cell #3)

**Fig. 5:**
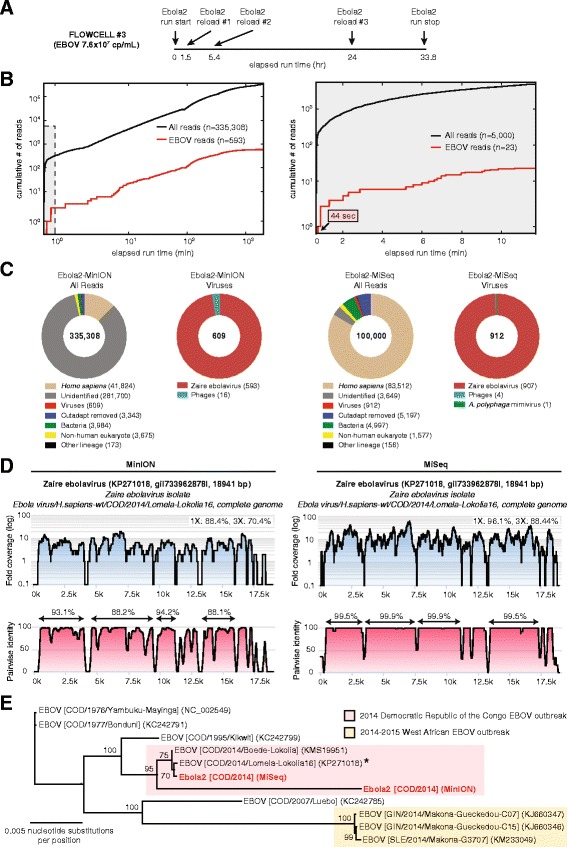
Metagenomic identification of EBOV from a clinical blood sample by nanopore sequencing and MetaPORE real-time bioinformatics analysis. Nanopore data generated from the Ebola2 library and sequenced on flow cell #3 were analyzed in real time using the MetaPORE bioinformatics analysis pipeline, and compared to corresponding Illumina MiSeq data. a Time line of nanopore sequencing runs on flow cell #3 with sample reloading, plotted as a function of elapsed time in hours since the start of flow cell sequencing. b Cumulative numbers of all sequenced reads (black line) and target viral reads (red line) from the nanopore run (left panel) or MiSeq run (right panel), plotted as a function of individual sequencing run time in minutes. c Taxonomic donut charts generated by real-time MetaPORE analysis of the nanopore reads (left panel) and post-run analysis of the MiSeq reads (right panel). The total number of reads analyzed is shown in the center of the donut. Note that given computational time constraints, only a subset of MiSeq reads (n = 100,000) was analyzed using MetaPORE. d Coverage and pairwise identity plots generated from nanopore (left panel) or MiSeq data (right panel) by mapping reads aligning to EBOV to the closest matching reference genome ((e), asterisk).  e Whole-genome phylogeny of EBOV. Representative EBOV genome sequences, including those from the 2014-2015 West Africa outbreak (tan) and 2014 DRC outbreak (pink), are included. Branch lengths are drawn proportionally to the number of nucleotide substitutions per position, and support values are shown for each node. Data were analyzed in MetaPORE on a 64-core Ubuntu Linux server using the January 2015 NT reference database.

To enable real-time analysis of nanopore sequencing data, we combined pathogen identification with monitoring and user-friendly web visualization into a real-time bioinformatics pipeline named MetaPORE. We tested MetaPORE by sequencing a nanopore library (Ebola2) constructed using the upgraded MAP-004 kit and corresponding to a whole blood sample from a patient with suspected Ebola hemorrhagic fever during the 2014 DRC outbreak. Four consecutive runs of the Ebola2 library on the same flow cell over 34 hr (Fig. 5a) yielded a total of 335,308 reads, of which 609 (0.18 %) aligned to EBOV (141 of 6009 or 2.3 %, of 2D pass reads), comparable to the 0.91 % achieved by Illumina MiSeq sequencing (Fig. 5c).

Notably, the first EBOV read was sequenced 44 s after data acquisition and correctly detected in ~3 min by MetaPORE (Fig. 5b, right panel; Additional file 3). The mapping of nanopore reads across the EBOV genome was relatively uniform with at least one read mapping to >88 % of the genome and areas of zero coverage also seen with much higher-coverage Illumina MiSeq data (Fig. 5d). The detection of EBOV by real-time metagenomic nanopore sequencing was confirmed by qRT-PCR testing of the clinical blood sample, which was positive for EBOV at an estimated titer of 7.64 × 10^7^ copies/mL. Phylogenetic analysis of the Ebola2 genome independently recovered by MinION nanopore and Illumina MiSeq sequencing revealed that nanopore sequencing alone was capable of pinpointing the correct EBOV outbreak strain and country of origin (Fig. 5e).

## Discussion

Unbiased point-of-care testing for pathogens by rapid metagenomic sequencing has the potential to transform radically infectious disease diagnosis in clinical and public health settings. In this study, we sought to demonstrate the potential of the nanopore instrument for metagenomic pathogen identification in clinical samples by coupling an established assay protocol with a new real-time sequence analysis pipeline. To date, high reported error rates (10–30 %) and relatively low throughput (<100,000 reads per flow cell) have hindered the utility of nanopore sequencing for analysis of metagenomic clinical samples [9, 11]. Prior work on infectious disease diagnostics using nanopore has focused on rapid PCR amplicon sequencing of viruses and bacteria [11], or real-time sequencing of pure bacterial isolates in culture, such as *Salmonella* in a hospital outbreak [12]. To our knowledge, this is the first time that nanopore sequencing has been used for real-time metagenomic detection of pathogens in complex, high-background clinical samples in the setting of human infections. Here, we also sequenced a near-complete viral genome to high accuracy (97–99 % identity) directly from a primary clinical sample and not from culture. As also demonstrated previously for the bacterium *Escherichia coli* K-12 [13], the CHIKV genome was assembled using only multiple overlapping, albeit error-prone, nanopore reads and without resorting to the use of a secondary platform such as an Illumina MiSeq for sequence correction (Fig. 2d).

Real-time sequence analysis is necessary for time-critical applications such as outbreak investigation [7] and metagenomic diagnosis of life-threatening infections in hospitalized patients [3, 4, 6]. NGS analysis for clinical diagnostics is currently performed after sequencing is completed, analogous to how PCR products were analyzed by agarose gel electrophoresis in the 1990s. Most clinical PCR assays to date have since been converted to a real-time format that reduces hands-on laboratory technician time and effort and decreases overall sample-to-answer turnaround times. Importantly, our nanopore data suggest that very few reads are needed to provide an unambiguous diagnostic identification, despite high individual per read error rates of 10–30 %. The ability of nanopore sequence analysis to identify viruses accurately to the species and even strain or genotype level is facilitated by the high specificity of viral sequence data, especially with the longer reads achievable by nanopore versus second-generation sequencing (Table 1, 452 bp; range 126–1477 bp).

Although the overall turnaround time for metagenomic sample-to-detection has now been reduced to <6 hr with nanopore sequencing, many challenges remain for routine implementation of this technology in clinical and public health settings. Improvements to make library preparation faster and more robust are critical, including automation and optimization of each step in the protocol. Standardized external and internal spike-in controls run in parallel will be needed to control for laboratory and carryover contamination. Here we looked only at clinical samples at moderate to high titers of 10^5^–10^8^ copies/mL, and the sensitivity of metagenomic nanopore sequencing at lower titers remains unclear at current achievable sequencing depths. Standard wash protocols also appear inadequate to prevent carryover contamination when reusing the same flow cell, as CHIKV reads were identified in the downstream Ebola1 sample sequence run. One solution may be to perform only one nanopore sequencing run per flow cell for clinical diagnostic purposes, akin to how individual disposable cartridges are used for clinical quantitative PCR testing on a Cepheid GenXpert instrument to prevent cross-contamination [39]. Another potential solution is to give unique barcodes to individual samples as part of a multiplexed sequencing run at the cost of added time and effort.

A key challenge with microbial identification by metagenomic nanopore sequencing is that the current accuracy of sparse nanopore reads is insufficient to allow confident species identification of bacteria, fungi, or parasites, which have much larger genomes and share more conserved genes than viruses. Indeed, distinct bacterial species are often defined by as little as 5 % genomic divergence and 1 % sequence divergence in highly conserved housekeeping genes such as 16S ribosomal RNA [40]. Of note, the majority of nanopore reads aligning to bacteria in this study likely originated from the inclusion of lambda phage DNA in the sequencing library, reagent contamination, or, for the Ebola virus samples, environmental contamination from sample collection in a rural hospital setting (Additional file 4: Table S3). Accurate identification of eukaryotic pathogens from sparse, error-prone nanopore reads also appears to be challenging (Additional file 4: Table S3). In addition, single-nucleotide resolution will likely be required for detection of antimicrobial resistance markers [41], which is difficult to achieve from relatively low-coverage metagenomic data [42]. These limitations can potentially be overcome in the future by target enrichment methods such as capture probes to increase coverage, improvements in nanopore sequencing technology, or more accurate base-calling and alignment algorithms for nanopore data [43, 44].

## Conclusions

Our results indicate that unbiased metagenomic detection of viral pathogens from clinical samples with a sample-to-answer turnaround time of <6 hr and real-time bioinformatics analysis is feasible with nanopore sequencing. We demonstrate unbiased, diagnostic identification of EBOV within ~3 min of sequence acquisition. This technology will be particularly desirable for enabling point-of-care genomic analyses in the developing world, where critical resources, including reliable electric power, laboratory space, and computational server capacity, are often severely limited. Importantly, MetaPORE, the real-time sequencing analysis platform developed here, is web-based and can be run on a laptop. As sequencing yield, quality, and turnaround times continue to improve, we anticipate that third-generation technologies such as nanopore sequencing will challenge clinical diagnostic mainstays such as PCR and transcription-mediated amplification testing, fulfilling the dream of an unbiased, point-of-care test for infectious diseases.

## Additional files

Additional file 1: Table S1. Optimization of word count for MegaBLAST alignment to the NT reference database. (XLSX 12 kb) Additional file 2: Table S2. MetaPORE performance according to system configuration and alignment mode. (XLSX 18 kb) Additional file 3:
**Movie.** MetaPORE real-time bioinformatics analysis and visualization (clip 0:19–10:13, 9.8 min). Detection of EBOV by metagenomic nanopore sequencing and real-time MetaPORE bioinformatics analysis. Raw FAST5 files are uploaded to the Metrichor cloud-based analytics platform for 2D base-calling (clip, right panel). After downloading from Metrichor, base-called FAST5 reads are collected in batches of 200 reads and automatically processed in real time by MetaPORE. Read counts corresponding to detected organisms (e.g. humans, viruses, bacteria, non-human eukaryotes) and viral species are displayed in donut plots that are updated each minute in real time (left panel). Note that the first EBOV read from the Ebola2 sequencing run is detected 3 min 6 s (3:26) after the start of sequence acquisition (0:19). (clip 10:21–11:51, 1.5 min). Web-based, interactive coverage map and pairwise identity plots, generated in real time by MetaPORE, enable zooming, highlighting of individual values, outputting of relevant statistical data, and exporting of the graphs in various formats. The plots shown in the movie correspond to the analyzed data after completion of nanopore sequencing. Data were analyzed in MetaPORE on a 64-core Ubuntu Linux server using the January 2015 NT reference database. (MP4 20493 kb) Additional file 4: Table S3. Taxonomic classification of non-human nanopore reads identified using MetaPORE. (XLSX 117 kb)

## Abbreviations

bp: base pair
cDNA: complementary DNA
Chik1: chikungunya virus, strain PR-S6 sample
CHIKV: chikungunya virus
DNA: deoxyribonucleic acid
DRC: Democratic Republic of the Congo
Ebola1: Ebola virus, strain Lomela-Lokolia16 sample
Ebola2: Ebola virus, strain Lomela-LokoliaB11 sample
EBOV: Ebola virus
Gb: gigabase pair
HCV: hepatitis C virus
HepC1: hepatitis C virus, genotype 1b sample
HTML: hypertext markup language
kb: kilobase pair
MAP: MinION Access Program
MetaPORE: a bioinformatics analysis pipeline for real-time pathogen identification and visualization from nanopore NGS data
MinION: nanopore sequencing platform developed by Oxford Nanopore, Inc
NCBI: National Center for Biotechnology Information
NGS: next-generation sequencing
nt: nucleotide
NT database: NCBI nucleotide collection database
qRT-PCR: quantitative reverse transcription polymerase chain reaction
RNA: ribonucleic acid
SURPI: sequence-based ultra-rapid pathogen identification, a bioinformatics analysis pipeline for pathogen identification from NGS data developed at UCSF
UCSF: University of California, San Francisco
dNTP: deoxynucleotide triphosphate
DTT: Dithiothreitol
SS III RT: Superscript III reverse transcriptase
